# Intracellular dynamics of ubiquitin-like 3 visualized using an inducible fluorescent timer expression system

**DOI:** 10.1242/bio.060345

**Published:** 2024-11-05

**Authors:** Yuka Terada, Kumi Obara, Yusuke Yoshioka, Takahiro Ochiya, Haruhiko Bito, Kunihiro Tsuchida, Hiroshi Ageta, Natsumi Ageta-Ishihara

**Affiliations:** ^1^ Department of Biomolecular Science, Faculty of Science, Toho University, Funabashi, Chiba 274-8510, Japan; ^2^ Department of Molecular and Cellular Medicine, Institute of Medical Science, Tokyo Medical University, Shinjyuku-ku, Tokyo 160-0023, Japan; ^3^ Department of Neurochemistry, Graduate School of Medicine, The University of Tokyo, Bunkyo-ku, Tokyo 113-0033, Japan; ^4^ Division for Therapies Against Intractable Diseases, Center for Medical Science, Fujita Health University, Toyoake, Aichi 470-1192, Japan

**Keywords:** Post-translational modification (PTM), Ubiquitin-like 3 (UBL3), Multivesicular bodies (MVBs), Fluorescent timers

## Abstract

Exosomes are small extracellular vesicles (sEVs) secreted via multivesicular bodies (MVBs)/late endosomes and mediators of cell-cell communication. We previously reported a novel post-translational modification by ubiquitin-like 3 (UBL3). UBL3 is localized in MVBs and the plasma membrane and released outside as sEVs, including exosomes. Approximately 60% of proteins sorted in sEVs are affected by UBL3 and localized in various organelles, the plasma membrane, and the cytosol, suggesting that its dynamic movement in the cell before entering the MVBs. To examine the intracellular dynamics of UBL3, we constructed a sophisticated visualization system via fusing fluorescent timers that changed from blue to red form over time with UBL3 and by its expression under Tet-on regulation. Intriguingly, we found that after synthesis, UBL3 was initially distributed within the cytosol. Subsequently, UBL3 was localized to MVBs and the plasma membrane and finally showed predominant accumulation in MVBs. Furthermore, by super-resolution microscopy analysis, UBL3 was found to be associated with one of its substrates, α-tubulin, in the cytosol, and the complex was subsequently transported to MVBs. This spatiotemporal visualization system for UBL3 will form a basis for further studies to elucidate when and where UBL3 associates with its substrates/binding proteins before localization in MVBs.

## Teaser

**Summary:** The visualization system of fusing fluorescent timers with ubiquitin-like 3 (UBL3), a post-translational modification factor under Tet-on regulation, demonstrated the intracellular dynamics of UBL3.

## INTRODUCTION

Exosomes are a specific class of small extracellular vesicles (sEVs) secreted from a variety of cell types into the extracellular environment. Multivesicular bodies (MVBs)/late endosomes contain intraluminal vesicles (ILVs). When MVBs fuse with the plasma membrane, ILVs are released outside the cell as exosomes (Colombo et al., 2014). The sEVs are known to play a role in intercellular communication, as they encapsulate proteins and RNA and transmit them to other cells under physiological and pathological conditions (Mathieu et al., 2019; van Niel et al., 2018). They are involved in neurodegenerative diseases, dystrophic disorders, and cancer cell proliferation, invasion, and metastasis (Hill, 2019; Lucotti et al., 2022), because they contain neurodegenerative disease-related proteins, such as amyloid beta, tau, α-synuclein, and prions (Fevrier et al., 2004; Kanmert et al., 2015; Lee et al., 2014; Rajendran et al., 2006; Vella et al., 2007), oncogenic proteins, and signaling molecules (Choi et al., 2015). Skeletal muscles also release sEV and play a role in muscle physiology (Rome et al., 2019). sEVs containing functional proteins that control the growth and morphogenesis of mesenchymal stem cells have therapeutic effects on various diseases (Xunian and Kalluri, 2020). For example, sEV is useful for the treatment of muscle atrophy including muscular dystrophy (Aminzadeh et al., 2018).

After synthesis, proteins undergo various post-translational modifications to regulate their localization, stability, and function (Ageta-Ishihara et al., 2013; Ageta and Tsuchida, 2019; Deribe et al., 2010). Regulatory mechanisms involving post-translational modifications are involved in various diseases etiologies; discovering new post-translational modifications will lead to the development of new therapeutic strategies (Ahearn et al., 2011). In recent years, new post-translational modifications, such as lactylation and serotonylation, have been discovered (Farrelly et al., 2019; Zhang et al., 2019).

Ubiquitin-like 3 (UBL3)/membrane-anchored ubiquitin-fold (MUB) is a highly conserved protein in filamentous fungi, plants, and animals (Downes et al., 2006). In *Arabidopsis thaliana*, MUBs have a CAAX (where C is cysteine, A is any aliphatic amino acid, and X is any amino acid) motif at their C-terminus, undergo prenylation, and are enriched in the plasma membrane. The human, mouse, *Xenopus*, *Drosophila*, and zebrafish MUBs are also prenylated *in vitro* (Downes et al., 2006). In addition, UBL3 mRNA is a housekeeping gene in various human tissues (Chang et al., 2011) and a marker for various cancers (Huang et al., 2012; Lee et al., 2018; Shi et al., 2021; Zhao et al., 2020). We previously reported that UBL3 was a post-translational modification factor and released as sEVs. Approximately 60% of sEVs proteins were sorted by UBL3 (Ageta et al., 2018; Ageta and Tsuchida, 2019). Among the 1241 potential substrates/binding proteins for UBL3 modification, 22 disease-related proteins showed variable localizations either in the plasma membrane, organelles, and cytosol. Conversely, when UBL3 localization was studied using light and electron microscopy, it was confined to MVBs/late endosomes and the plasma membrane (Ageta et al., 2018), indicating that substrates for UBL3 modification and UBL3 do not colocalize completely within the cells. Therefore, UBL3 may exhibit dynamic subcellular localizations before being transported to MVBs.

To accurately track the intracellular dynamics of UBL3 after synthesis, it is necessary to express UBL3 created by fusing a fluorescent protein for a short period of time. Therefore, we used a Tet-on system to control inducible gene expression. Moreover, it is important to eliminate the possibility of slight leaky expression of a fluorescent protein in the absence of an inducer. Fluorescent timers (FTs) are fluorescent proteins that visualize spatiotemporal molecular events via changing their fluorescence from blue to red form over time (Terskikh et al., 2000). This change is caused by the maturation of the fluorescent protein over time and is thought to result from chromophore oxidation. Monomeric FTs have been developed to avoid the artificial localization of fluorescent proteins in cells due to aggregation (Subach et al., 2009; Tsuboi et al., 2010). FTs are used to elucidate biological events that change over time in cells, organelles, and proteins mobilization, contributing to our understanding of gene transcription (Bending et al., 2018), mitochondrial turnover (Hernandez et al., 2013), β-cell neogenesis and maturation (Miyatsuka et al., 2014), and protein trafficking (Subach et al., 2009).

To clarify the post-translational translocation of UBL3, we developed a system to visualize its spatiotemporal localization in living cells via controlling the expression of monomeric FT-fused UBL3 combined with the Tet-on system. Our data demonstrated that UBL3 was initially dispersed throughout the cytosol after synthesis. It was localized to MVBs and the plasma membrane, and ultimately, UBL3 showed predominant accumulation within MVBs. Moreover, by super-resolution microscopy analysis, we showed that UBL3 was bound to its substrate, α-tubulin, in the cytosol and subsequently transported to MVBs. The spatiotemporal visualization system for UBL3 presented here will be useful for studies aimed at elucidating the trafficking pathways of UBL3 substrates/binding proteins before localization in MVBs.

## RESULTS

### Design and application of a system for spatiotemporal visualization of UBL3 post-translational translocation

**Fig. 1.**
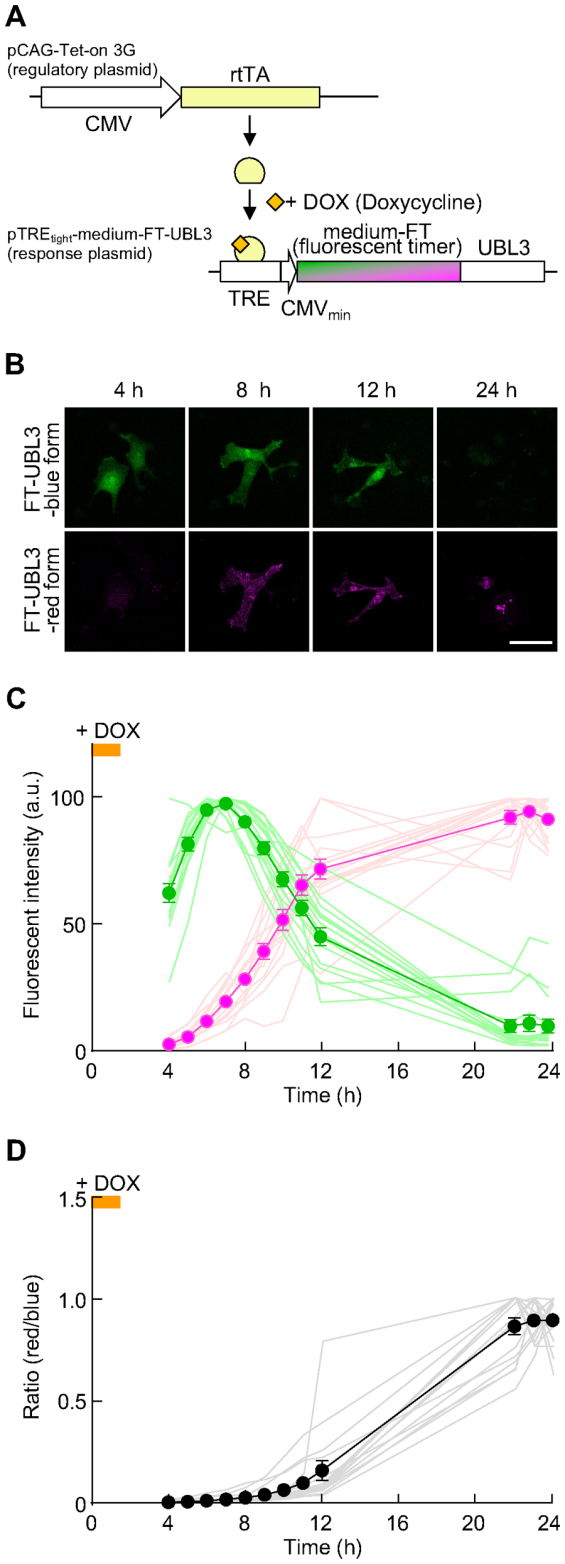
**Design and construction of a system for spatiotemporal visualization of UBL3 localization.** (A) Schematic diagram of plasmids and the Tet-On inducible system. The pCAG-Tet-on 3G is a regulatory plasmid containing DOX-inducible reverse tetracycline transactivator (rtTA) under the cytomegalovirus (CMV) promoter. The pTRE_tight_-medium-FT-UBL3 is a response plasmid containing the expression cassette of UBL3 fused to the C-terminus of the medium-fluorescent timer (FT) under the promoter comprising tetracycline response element (TRE) and minimal cytomegalovirus promoter (CMV mini). The cells were transfected with pCAG-Tet-on 3G plasmid and pTRE_tight_-medium-FT-UBL3 plasmid. When DOX was added to the medium, it bound to rtTA, which in turn was bound to TRE and induces medium-FT-UBL3 gene expression. When DOX was washed out, rtTA was dissociated from TRE, and gene expression was terminated. (B) Representative sum intensity projection sequential images of identical MDA-MB-231 cells transfected with FT-UBL3 after 4, 8, 12, and 24 h of DOX treatment. Green pseudo-color, blue form of FT-UBL3. Magenta pseudo-color, red form of FT-UBL3. Scale bar: 50 µm. (C,D) Time changes of the blue (green line and circles) and red (magenta line and circles) sum fluorescence intensities (C) and ratio value (red form/blue form) (D) of FT-UBL3-expressing cells. The orange line indicates DOX induction time window. Data are presented as mean±s.e.m. Light colored lines depict data from individual cell (*n*=16 cells from three independent experiments).

Monomeric FTs have three variants (slow-FT, medium-FT, and fast-FT) with different chromophore maturation rates; the transport pathway of lysosome-associated membrane protein type 2A to lysosomes was visualized using medium-FT (Subach et al., 2009). To elucidate the intracellular dynamics of UBL3, we constructed medium-FT-UBL3 via fusing medium-FT to the N-terminus of UBL3, since UBL3 has a CAAX motif required for plasma membrane localization (Downes et al., 2006) and two cysteine residues involved in UBL3 modification at the C-terminal region (Ageta et al., 2018). Furthermore, medium-FT-UBL3 was combined with the Tet-on system (Inoue et al., 2015; Inoue et al., 2019) to spatiotemporally track UBL3 expression in a limited time (Fig. 1A). MDA-MB-231 cells, a human breast cancer line, has been used to elucidate UBL3 as a post-translational modification factor (Ageta et al., 2018), were treated with doxycycline (DOX) for 1.5 h. The fluorescence shift of medium-FT-UBL3 from blue to red form was measured over time from 4 h, the time when the fluorescence of FT-UBL3 became visible, to 24 h after DOX treatment (Fig. 1B,C). The relative brightness value of the blue form reached its maximum 7 h after DOX treatment and then gradually declined by 24 h. The relative value of brightness of the red form reached its maximum at approximately 22–24 h after DOX treatment (Fig. 1B,C). Furthermore, the ratio value of red form/blue form indicated that the conversion to the red form reached a plateau 22 h after DOX treatment (Fig. 1D). Overall, we provided a spatiotemporal visualization system for tracing UBL3 localization that could capture UBL3 subcellular dynamics 24 h after DOX treatment.

### UBL3 is transported from the cytosol to MVBs/late endosomes and the plasma membrane and finally accumulates in MVBs

**Fig. 2.**
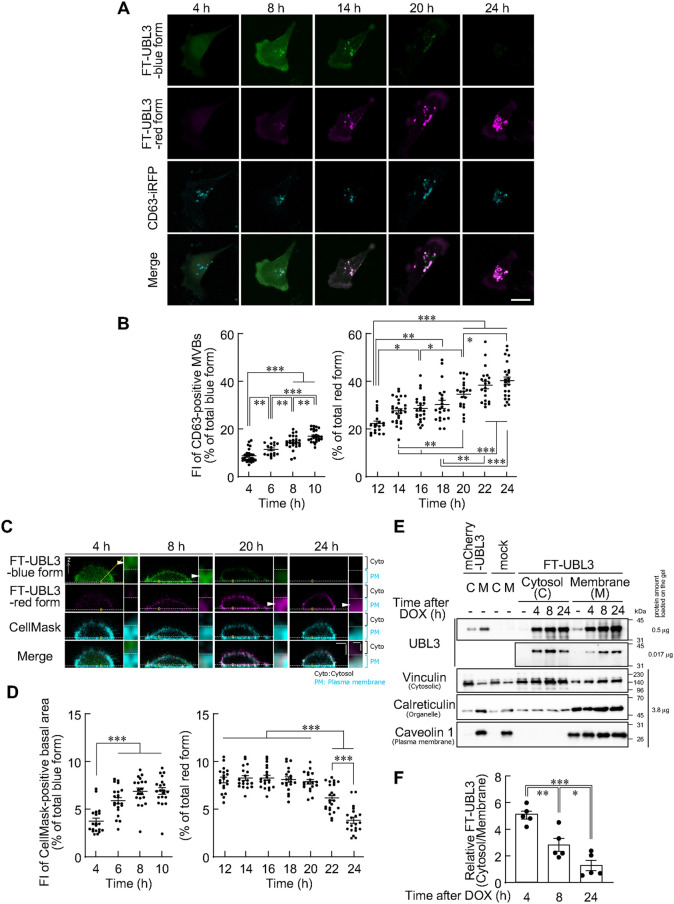
**The subcellular route of UBL3 transported to multivesicular bodies.** (A) Representative maximum intensity projection images of different MDA-MB-231 cells transfected with FT-UBL3 plus CD63-iRFP670 (a multivesicular body marker) after 4, 8, 14, 20, and 24 h of DOX treatment. Green pseudo-color, blue form of FT-UBL3. Magenta pseudo-color, red form of FT-UBL3. Scale bar: 20 µm. (B) Quantitative analysis of the blue (after 4, 6, 8, and 10 h of DOX treatment) and red (after 12, 14, 16, 18, 20, 22, and 24 h of DOX treatment) fluorescence intensities of FT-UBL3-expressing cells in CD63-positive MVBs. (C) Representative vertical cross-sections of different MDA-MB-231 cells transfected with FT-UBL3 and labeling of the plasma membrane using the CellMask Deep Red plasma membrane stain. Yellow squares indicate cropped areas. The white dashed lines indicate the boundary between the cytosol and the plasma membrane defined by the CellMask plasma membrane stain. Arrowheads indicate the localization of FT-UBL3. Scale bars: 5 µm and 0.3 µm. (D) Quantitative analysis of blue (after 4, 6, 8, and 10 h of DOX treatment) and red (after 12, 14, 16, 18, 20, 22, and 24 h of DOX treatment) fluorescence intensities of FT-UBL3-expressing cells in the CellMask-positive basal area. (E) Western blot analysis of cytosolic and membrane fractions of FT-UBL3 expressing MDA-MB-231 cells after 4, 8, and 24 h of DOX treatment using antibodies against Vinculin (cytosolic marker), Calreticulin (organelle membrane marker) and Caveolin 1 (plasma membrane marker). (F) Quantitative analysis of the ratio of FT-UBL3 protein in the cytosolic fraction relative to the membrane fraction, with 0.017 μg of protein. Data are presented as mean±s.e.m. Dots indicate data from individual cells (*n*=17–28 cells from more than three independent experiments) (B,D) or individual experiments (*n*=5) (F). One-way analysis of variance with Tukey's multiple comparison test. *P*-values are shown at the top of the graphs only for the time periods in which significant differences were found for B, D and F. ***P<0.001, **P<0.01, *P<0.05.

The intracellular dynamics of UBL3 after translation was then determined. We created a plasmid with cluster of differentiation 63 (CD63), a marker for MVBs, fused to the N-terminus of the near-infrared fluorescent protein iRFP670 (CD63-iRFP670), and transfected MDA-MB-231 cells with FT-UBL3 and CD63-iRFP670. The fluorescence intensity of the blue form was higher than that of the red form 4–10 h after DOX treatment, and that of the red form became higher than that of the blue form at 12–24 h after DOX treatment (Fig. 1). We observed the localization of FT-UBL3 in blue form at 4–10 h and in red form at 12–24 h after DOX induction. We analyzed the fluorescence intensity of FT-UBL3 in the CD-63 positive (MVBs). We found that FT-UBL3 in blue form localized to the cytosol 4 h after DOX induction, and the fluorescence intensity of FT-UBL3 in blue form gradually increased in MVBs from 4 h to 10 h after DOX induction (Fig. 2A,B). FT-UBL3 in red form gradually increased in MVBs from 12 h to 24 h after DOX induction (Fig. 2A,B).

Next, to analyze the plasma membrane localization of FT-UBL3 after synthesis, we visualized the plasma membrane in MDA-MB-231 cells using CellMask, a reagent specifically designed for plasma membrane staining (Efremov et al., 2019), and quantified the fluorescence intensity of FT-UBL3 in the CellMask-positive regions (the plasma membrane). FT-UBL3 in blue form was localized to the cytosol 4 h after DOX induction and the fluorescence intensity of FT-UBL3 in blue form gradually increased in the plasma membrane from 4 h to 10 h after DOX induction (Fig. 2C,D). FT-UBL3 in red form decreased in the plasma membrane from 20 h to 24 h after DOX induction (Fig. 2C,D). Where the blue form of FT-UBL3 showed cytosolic localization 4 h after DOX induction but the red form did not, suggesting that FT-UBL3 was transported from the cytosol either to the MVBs and plasma membrane. To quantify the cytosolic localization of FT-UBL3, we detected the amount of FT-UBL3 in both cytosolic and membrane fractions at 4, 8, and 24 h after DOX induction. The relative amount of FT-UBL3 in the cytosolic fraction was the highest at 4 h after DOX induction and decreased over time (Fig. 2E,F). There results indicated that UBL3 initially exhibited a diffuse distribution after synthesis, subsequently localized to both MVBs and the plasma membrane, and ultimately showed predominant accumulation in MVBs.

### Analyses of the changes of spatiotemporal localization mutants lacking UBL3 modification activity

**Fig. 3.**
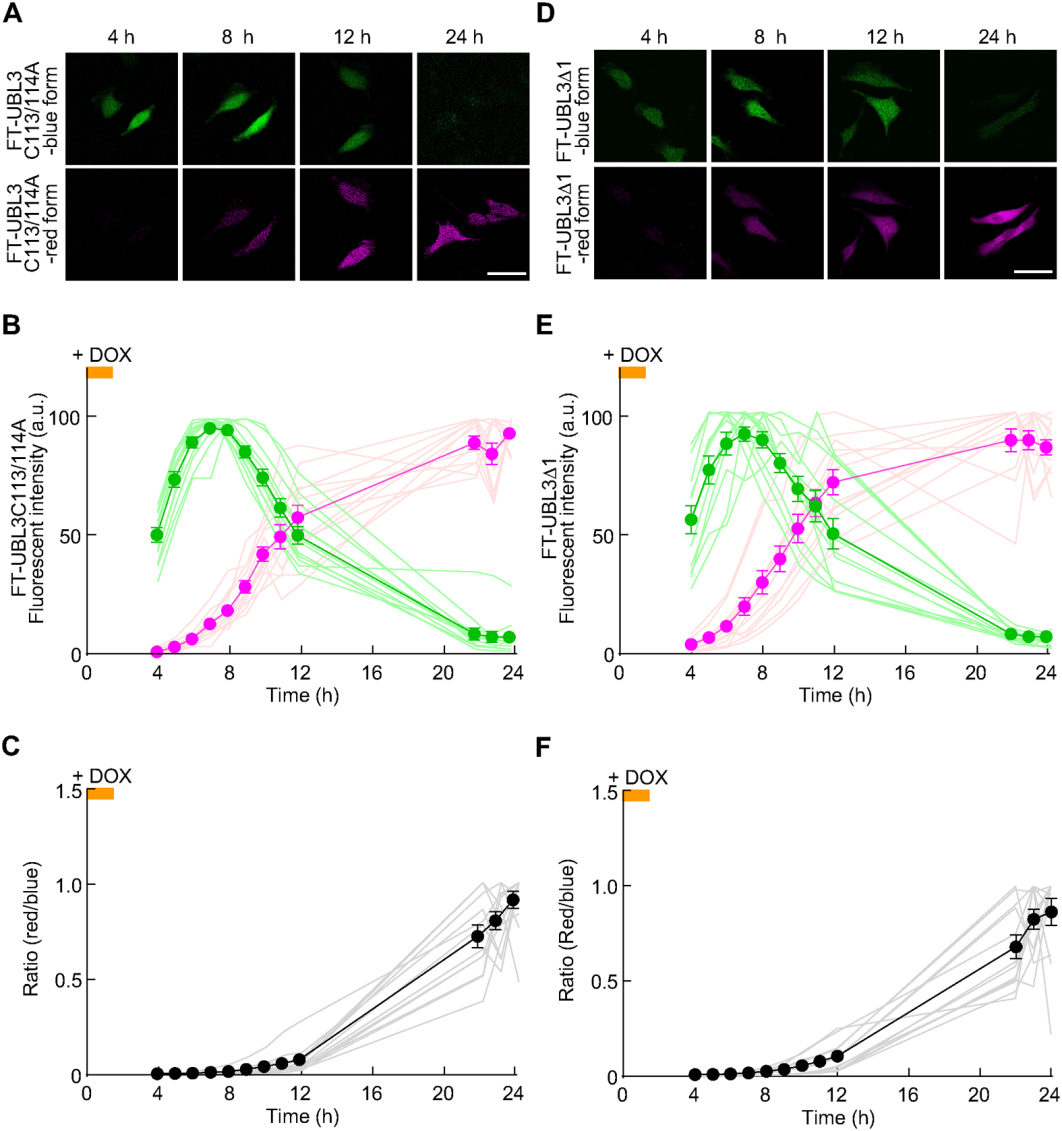
**Characterization of a system for spatiotemporal dynamics of UBL3 mutants, C113/114A (lacking both UBL3 modification activity and membrane localization) and UBL3Δ1 (retaining membrane localization, lacking UBL3 modification activity).** (A,D) Representative sequential images of identical MDA-MB-231 cells transfected with FT-UBL3C113/114A (A) and FT-UBL3Δ1 (D) after 4, 8, 12, and 24 h of DOX treatment. Scale bars: 50 µm. (B,E) Time changes of the blue (green line and circles) and red (magenta line and circles) sum fluorescence intensities of FT-UBL3 C113/114A (B) and FT-UBL3Δ1 expressing cells (E). (C,F) Time changes in the ratio of red form/blue form of FT-UBL3 C113/114A (C) and FT-UBL3Δ1 expressing cells (F). The orange line indicates DOX induction time window. Data are presented as mean±s.e.m. Light colored lines depict data from individual cell (*n*=12 cells from more than three independent experiments).

Next, we analyzed and determined the necessity of UBL3 modification activity, which is the attachment to other proteins, for plasma membrane and/or MVBs localization. In our previous study, we reported two mutants; one lost both UBL3 modification activity and plasma membrane localization (UBL3C113/114A), and the other mutant lost UBL3 modification activity but retained plasma membrane localization (UBL3Δ1) (Ageta et al., 2018). We created FT-UBL3 C113/114A and FT-UBL3Δ1 to visualize the spatiotemporal localization. The fluorescence change from the blue to red form of FT-UBL3C113/114A was traced from 4 to 24 h. Fluorescence of the blue form reached its maximum at 7 h after DOX induction, and its intensity gradually decreased and was undetectable at 24 h after DOX induction (Fig. 3A,B). In contrast, the red fluorescence gradually increased after DOX induction (Fig. 3A,B). The ratio of red form/blue form gradually increased over 24 h after DOX treatment (Fig. 3C). The same analysis of FT-UBL3Δ1 revealed that fluorescence of the blue form reached a maximum 8 h, and then gradually decreased and was undetectable at 24 h after DOX induction (Fig. 3D,E). In contrast, the red form gradually increased, reaching a maximum at 22 h after and plateauing 24 h after DOX induction (Fig. 3D,E). The ratio of red form/blue form reached a maximum 24 h after DOX induction (Fig. 3F). These results indicated that even when the fluorescent timer was fused to two UBL3 mutants, no significant differences were observed in the timing of the shift from the blue to red form.

### UBL3 conjugation activity is required for UBL3 transport to MVBs

**Fig. 4.**
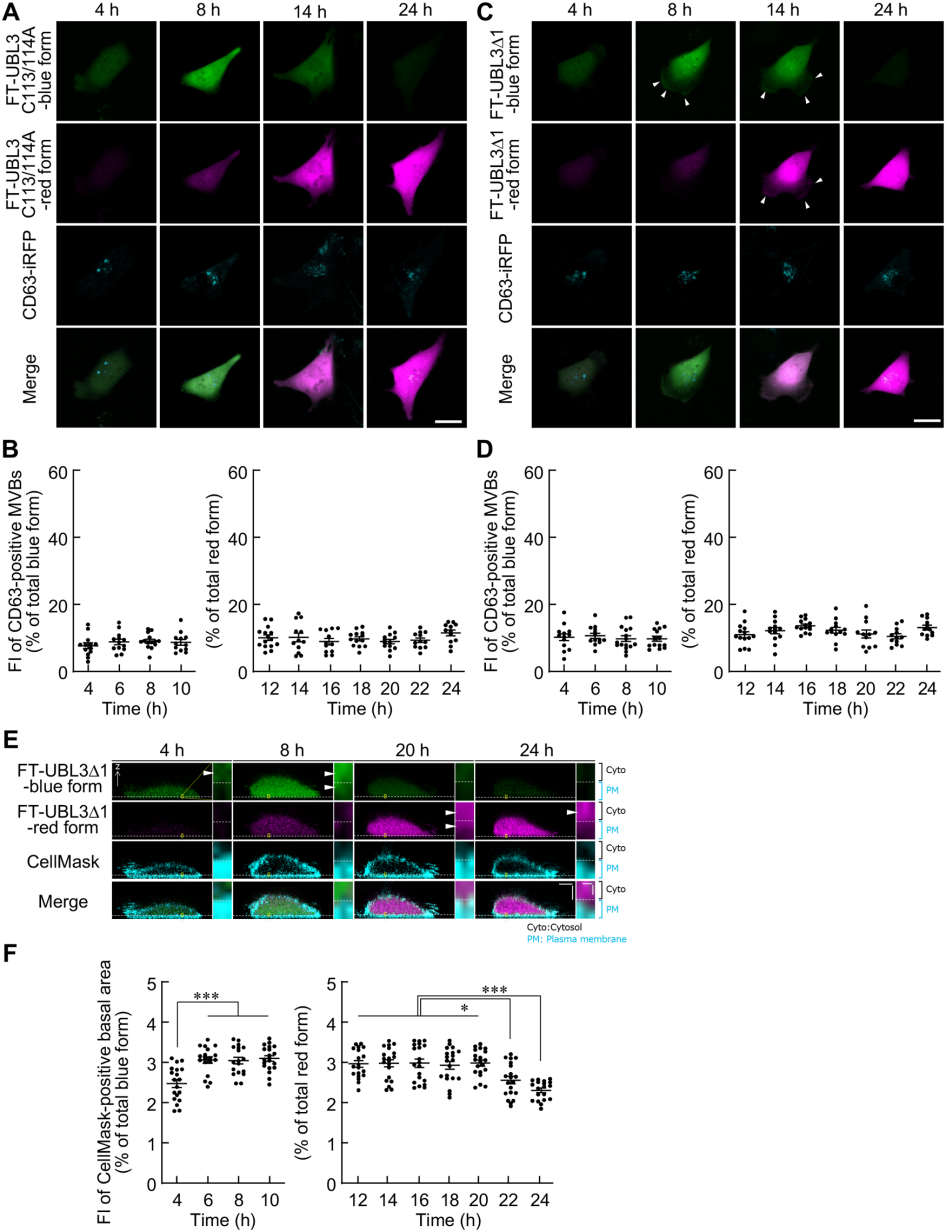
**The spatiotemporal localization of UBL3C113/114A (lacking both UBL3 modification activity and membrane localization) and UBL3Δ1 (retaining membrane localization, lacking UBL3 modification activity).** (A,C) Representative maximum intensity projection images of different MDA-MB-231 cells transfected with FT-UBL3C113/114A (A) and FT-UBL3Δ1 (C) plus CD63-iRFP670 (a multivesicular body marker) after 4, 8, 14, and 24 h of DOX treatment. Green pseudo-color, blue form of FT-UBL3C113/114A and FT-UBL3Δ1. Magenta pseudo-color, red forms of FT-UBL3C113/114A and FT-UBL3Δ1. Arrowheads indicate the cell periphery. Scale bars: 20 µm. (B,D) Quantitative analysis of the blue (after 4, 6, 8, and 10 h of DOX treatment) and red (after 12, 14, 16, 18, 20, 22, and 24 h of DOX treatment) fluorescence intensities of FT-UBL3C113/114A and FT-UBL3Δ1-expressing cells in CD63-positive MVBs. (E) Representative vertical cross-sections of different MDA-MB-231 cells transfected with FT-UBL3Δ1 and labeling of the plasma membrane using the CellMask Deep Red plasma membrane stain. Yellow squares indicate cropped areas. The white dashed lines indicate the boundary between the cytosol and the plasma membrane defined by the CellMask plasma membrane stain. Arrowheads indicate the localization of FT-UBL3Δ1. Scale bars: 5 µm and 0.3 µm. (F) Quantitative analysis of the blue (after 4, 6, 8, and 10 h of DOX treatment) and red (after 12, 14, 16, 18, 20, 22, and 24 h of DOX treatment) fluorescence intensities of FT-UBL3Δ1-expressing cells in the CellMask-positive plasma membrane. Data are presented as mean±s.e.m. Dots indicate data from individual cells (*n*=12–20 cells from more than three independent experiments). One-way analysis of variance with Tukey's multiple comparison test. *P*-values are shown at the top of the graphs only for the time periods in which significant differences were found for B, C, D, F, G, and H. ***P<0.001, **P<0.01, and *P<0.05.

To examine the relationship between the plasma membrane and/or MVB localization of UBL3 and UBL3 modification activity, FT-UBL3C113/114A and CD63-iRFP670, a marker for MVBs, were co-expressed in MDA-MB-231 cells. The results showed that FT-UBL3C113/114A did not localize either to the MVB or plasma membrane 4–24 h after DOX induction (Fig. 4A,B). Furthermore, analyzing the subcellular localization of FT-UBL3Δ1, which retained its plasma membrane localization but lost UBL3 modification activity, revealed that the fluorescence intensity of the blue form of FT-UBL3Δ1 gradually increased in the plasma membrane at 4–6 h after DOX induction, whereas its fluorescence in MVBs did not change (Fig. 4C–F). Fluorescence of the red form of FT-UBL3Δ1 was not detected in MVBs at any time course (Fig. 4C,D). In contrast, its fluorescence was retained in the plasma membrane 12–20 h after DOX induction and then weakened from 20 to 24 h after DOX treatment (Fig. 4E,F). The plasma membrane localization of FT-UBL3Δ1 at 4 and 24 h after DOX treatment was almost the same (Fig. 4E,F). Taken together, our data demonstrate that UBL3 modification activity is important for localization to MVBs.

### Spatiotemporal visualization system for UBL3 captures the association of UBL3 with α-tubulin, one of the UBL3 substrates, and its transport to MVBs

**Fig. 5.**
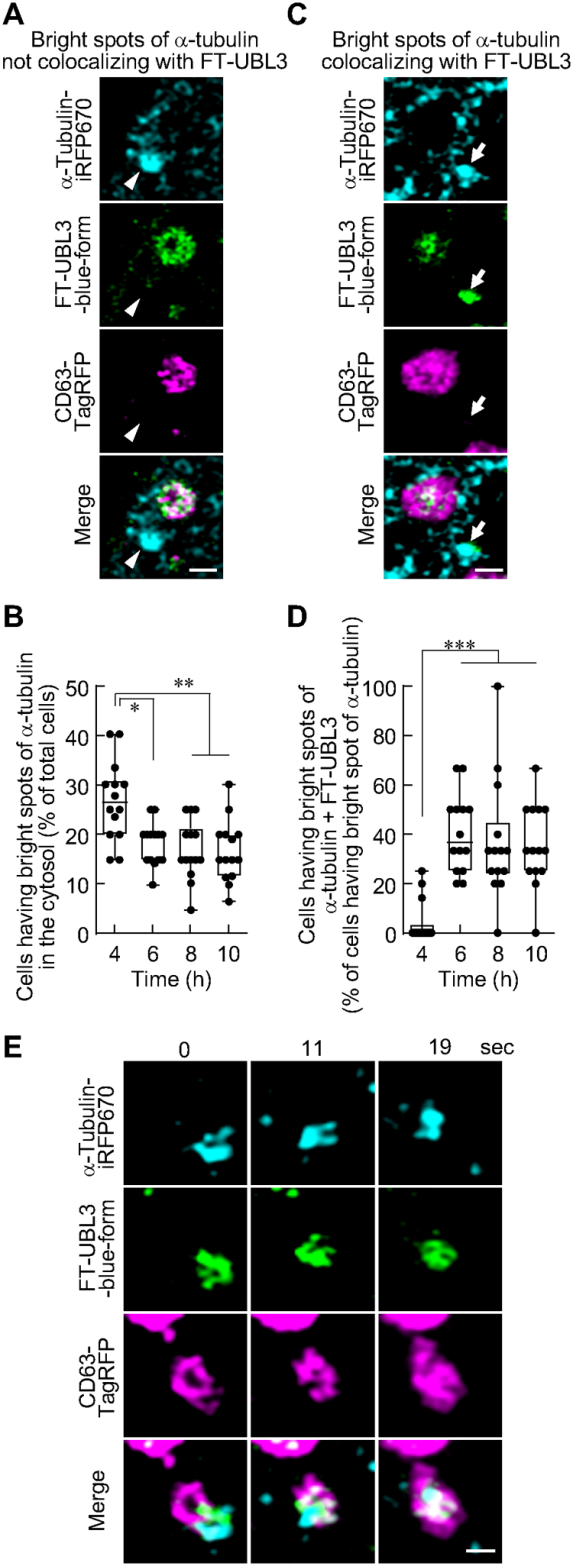
**Association of UBL3 and α-tubulin, its substrate, in the cytosol and transport to MVBs.** (A) Representative super-resolution images show bright spots of α-tubulin-iRFP670 with a long diameter of 200–800 nm in the cytosol, the blue form of FT-UBL3, and TagRFP-CD63 (a MVB marker). Arrowheads indicate bright spots of α-tubulin not colocalizing with FT-UBL3. Scale bar: 1 µm. (B) Quantitative analysis of the percentage of cells with bright spots of α-tubulin with a long diameter of 200–800 nm in the cytosol. (C) Representative super-resolution images show bright spots of α-tubulin-iRFP670 colocalized with the blue form of FT-UBL3 and TagRFP-CD63. Arrows indicate bright spots of α-tubulin colocalizing with FT-UBL3. Scale bar: 1 µm. (D) Quantitative analysis of the percentage of cells with bright spots of α-tubulin co-localized with FT-UBL3 in the cytosol divided by the number of cells that have bright spots of α-tubulin in the cytosol. (E) Representative super-resolution time-lapse images to track the bright spot of α-tubulin colocalized with the blue form of FT-UBL3 and TagRFP-CD63. Scale bar: 500 nm. Data are presented as min to max. Dots indicate data from independent experiments (*n*=14 independent experiments). Kruskal–Wallis with Dunn's test. *P*-values are shown at the top of the graphs. ***P<0.001, **P<0.01, and *P<0.05.

Finally, to verify whether the UBL3 visualization system was useful to assess when and where UBL3 and its substrates associate and are transported to MVBs, we visualized UBL3 and one of its substrates, α-tubulin, a target of UBL3 modification (Ageta et al., 2018). Super-resolution live imaging of FT-UBL3 and α-tubulin-iRFP670 (Zhang et al., 2023) revealed that bright spots of α-tubulin, with diameters ranging from 200–800 nm, appeared in the cytosol 4 h after DOX induction (Fig. 5A,B). From 4 to 6 h after DOX induction, the percentage of cells with bright spots of α-tubulin not colocalizing with FT-UBL3 in the cytosol decreased and were retained 6–10 h after DOX induction (Fig. 5B). Furthermore, 6 h after DOX induction, UBL3 co-localized with bright spots of α-tubulin in the cytosol (Fig. 5C,D). The percentage of cells with bright spots of α-tubulin co-localized with FT-UBL3 in the cytosol increased from 4 to 6 h after DOX induction and remained constant until 10 h after DOX induction (Fig. 5D). In 14 experiments, we observed seven spots of α-tubulin colocalized with FT-UBL3 in close proximity to MVBs from 6 to 10 h after DOX induction. All spots were incorporated into MVBs within a minute (Fig. 5E). These data indicated that UBL3, after its synthesis, was associated with one of its substrates, α-tubulin, in the cytosol and transported to MVBs subsequently.

## DISCUSSION

Ubiquitin modifications within the UBL family have been extensively studied. Cellular responses of substrate proteins undergoing ubiquitination are regulated by decoder molecules that recognize the ubiquitin chain (Komander and Rape, 2012; Kwon and Ciechanover, 2017). Decoder molecules detect the spatial and temporal aspects of ubiquitin modifications and translate this information into various biological processes (Grice and Nathan, 2016; Scott et al., 2015), such as proteolysis by proteasomes, DNA damage repair, autophagy, and immune response (Collins and Goldberg, 2017; Lim and Yue, 2015; Pickart and Fushman, 2004; Yang et al., 2009). In the current study, it became apparent that UBL3 requires its modification activity to be transported to MVBs (Figs 2, 4). UBL3 may associate with decoder molecules to regulate UBL3 trafficking to MVBs and the fate of potential substrates of UBL3 modification.

Ubiquitin creates hybrid chains with ubiquitin-like modifiers, such as small ubiquitin-like modifiers (SUMO), neural cell expressed, developmentally downregulated gene 8 (NEDD8), and interferon-stimulated gene 15 (ISG15) (Morris and Garvin, 2017; Wang et al., 2017; Yau and Rape, 2016). Ubiquitin-SUMO hybrid chains degrade SUMOylated substrates (Uzunova et al., 2007). MUB/UBL3 has been reported to show plasma membrane localization in *Arabidopsis* plants (Downes et al., 2006) and is involved in ubiquitination (Dowil et al., 2011; Lu et al., 2016). As UBL3 is released outside the cell as an exosome via MVBs (Ageta et al., 2018), and UBL3 localizes to MVBs and the plasma membrane, and eventually, MVB localization becomes predominant; it may be cross-linked with the ubiquitination pathway during transport from the plasma membrane to MVB. It could alter the fate of plasma membrane proteins that serve as substrates for UBL3 modification and release them as exosomes.

This study revealed that the synthesized UBL3 exhibited diffuse distribution throughout the cytosol before transitioning to localization within both MVBs and the plasma membrane, and ultimately, UBL3 resided predominantly within MVBs. Two types of protein synthesis are known: via free ribosomes in the cytosol, and via ribosomes bound to the endoplasmic reticulum (Alberts et al., 2022; Palade, 1975). In our previous study, UBL3 did not co-localize with calnexin, a marker for endoplasmic reticulum (Ageta et al., 2018); hence, UBL3 could be synthesized from free ribosomes at least in the cytosol and transported to the plasma membrane and MVBs without endoplasmic reticulum-mediated transport. In this study, we showed that the plasma membrane localization of FT-UBL3 decreased from 20 to 24 h after DOX induction (7.84%±0.23 at 20 h, 3.85%±0.30 at 24 h, Fig. 2D), while the MVB localization of FT-UBL3 increased (34.7%±1.30 at 20 h, 40.4%±1.49 at 24 h, Fig. 2B). In contrast, the plasma membrane localization of FT-UBL3Δ1, the mutant lacking UBL3 modification activity, decreased from 20 to 24 h after DOX induction (2.98%±0.07 at 20 h, 2.31%±0.05 at 24 h, Fig. 4F) and did not localize in MVBs (Fig. 4C,D). Furthermore, we previously demonstrated that the majority of UBL3 released outside the cells was included in the sEV fraction, which contains exosomes derived from MVBs, whereas a small fraction of UBL3 and UBL3Δ1 was included in the fraction derived from the plasma membrane as EVs using the method for isolating EVs (Ageta et al., 2018). Therefore, UBL3 on the plasma membrane may be predominantly transported to MVBs and partially released directly from the plasma membrane as EVs. If UBL3 localized to the plasma membrane is transported to MVBs, the following occurs: Plasma membrane proteins are transported to late endosomes via the canonical endosomal pathway, which is the route from the plasma membrane to MVBs/late endosomes via the early endosomes (Maxfield and McGraw, 2004; Mellman, 1996). To determine the UBL3 transport route from the plasma membrane to MVBs via the canonical endosomal pathway or a different route, in the future, creating mutants that retain UBL3 modification activity and plasma membrane localization, but do not exhibit MVBs localization, could further elucidate the pathway from the plasma membrane to MVBs and/or from the cytosol to MVBs. Alternatively, the above question may be solved by developing a new inhibitor for UBL3 modification activity or the identification of molecules involving its transport to the MVBs.

## MATERIALS AND METHODS

### Cell culture

MDA-MB-231-luc-D3H2LN breast cancer cells provided by Dr. Yusuke Yoshiok and Takahiro Ochiya (MDA-MB-231 cells) were cultured in Roswell Park Memorial Institute (RPMI) 1640 medium (11875-093, GIBCO) with 10% heat-inactivated fetal bovine serum (FBS) (SH30910.03, Cytiva). Cultures were incubated in 5% CO_2_ at 37°C.

### Plasmid construction

The pTRE-Medium-fluorescent timer (FT) was a gift from Vladislav Verkhusha (Addgene plasmid #31914; http://n2t.net/addgene:31914; RRID:Addgene_31914). Medium-FT fused to mouse UBL3 was subcloned into pTRE_tight_ (Inoue et al., 2019). Various mutants of FT-UBL3 were created using site-directed mutagenesis. The pCAG-Tet-on 3G was as described previously (Inoue et al., 2015). CD63 from pCT-CD63-GFP (CYTO120-PA-1, System Biosciences) and TagRFP, or iRFP670 were subcloned into pcDNA3. pTubulin-iRFP670 was a gift from Kiryl Piatkevich (Addgene plasmid # 197237; http://n2t.net/addgene:197237; RRID:Addgene_197237).

### Live imaging

MDA-MB-231 cells were plated onto a poly-D-Lysine-coated glass-bottom 35 mm dish (P35GC-1.5-14-C, Matteck) at a density of 4×10^6^ cells/dish. After 48 h, the medium was replaced with a Phenol Red-free imaging medium (RPMI 1640 medium, 11835030, GIBCO) with 10% FBS and the cells were transfected with plasmids using Lipofectamine 2000 (11668030, Thermo Fisher Scientific). After 24 h, the cells were treated with 10 ng/ml doxycycline (B-0801, Echelon) for 1.5 h before imaging within a stage-top incubator (TOKAI HIT, STX) kept in 5% CO_2_ at 37°C. The plasma membrane was stained with the CellMask Orange Plasma membrane Stain (C10045, Thermo Fisher Scientific) for 10 min at 5% CO_2_ at 37°C before each time point following to the manufacturer's protocol. Confocal and super-resolution images were taken with a 20× or 63× objective lens (NA 0.8 or 1.4) on a confocal laser microscope (LSM900 with Airyscan 2, Carl Zeiss). We analyzed images captured within 20 min (Figs 1 and 3) after each time point or 30 min (Figs 2, 4, and 5) before and after each time point. We sequentially acquired images of ten or more cells in each experiment and analyzed cells that could be tracked at all time points, as shown in Figs 1 and 3. In Figs 2 and 4, before image acquisition, we conducted a preliminary examination of cells across the field. Subsequently, we registered and acquired 4-channel images (Figs 2A and 4A,C) or 3-channel images (Figs 2C and 4E) of 2.53 s per frame with a z-stack of 50–60 images of cells with average brightness and localization. In Fig. 5, we counted 17–35 cells per time point using a 63× objective, zoom 5 in airy scan mode and calculated the percentage of cells with bright spots of α-tubulin in the cytosol or UBL3 co-localized with bright spots of α-tubulin with a long diameter of 200–800 nm in the cytosol. Representative maximum intensity projection images of CD63-iRFP670 were obtained via background subtraction using a rolling ball algorithm with a radius of 50. Fluorescence intensity was measured using Fiji software (ImageJ). Relative fluorescence intensities of the blue and red forms and the ratio of red form/blue form of FT-UBL3 were normalized using a maximum value of 100%. The percentage of blue or red forms of FT-UBL3 was calculated as follows: (%)=(summation of blue or red forms of the FT-UBL3 signal in each area in each confocal section) / (summation of blue or red forms of the FT-UBL3 signal in the total cell area in each confocal section)×100. The ratio (red form / blue form) was calculated as follows: ratio=(summation of red forms of the FT-UBL3 signal in each cell area in each confocal section) / (summation of blue forms of the FT-UBL3 signal in each cell area in each confocal section). The analysis area must be perpendicular to the focal plane for quantifying the relative fluorescence intensities in the plasma membrane therefore, ROI was set in the CellMask-positive basal area.

### Biochemical analysis

MDA-MB-231 cells were plated in a 60 mm dish (430166, CORNIG) at a density of 0.6×10^6^ cells/dish. After 48 h, the cells were transfected with plasmids using Lipofectamine 2000 (11668030, Thermo Fisher Scientific). After 24 h, the cells were treated with 10 ng/ml DOX (B-0801, Echelon) for 1.5 h before biochemical analysis. The cytosolic and membrane fractions were separated using the Mem-PER Plus kit (89842, Thermo Fisher Scientific) with 3×Halt Protease and Phosphatase Inhibitor Cocktail (78440, Thermo Fisher Scientific). These samples were boiled in 2× sample buffer (100 mM Tris–HCl at pH 6.8, 4% SDS, 20% glycerol, 0.01% Bromophenol Blue and 10% 2-mercaptoethanol). Protein concentration in each fraction was measured using a BCA Protein Assay kit (23227, Thermo Fisher Scientific). Anti-UBL3 (14100-1-AP, Proteintech, 1:1000), anti-Vinculin (EPR8185, Abcam, 1:1000), anti-Carleticulin (#2891, Cell Signaling Technology, 1:500), anti-Caveolin 1 (610407, BD Biosciences, 1:500) and HRP-conjugated secondary antibody (anti-rabbit IgG, #7074S, Cell Signaling Technology, 1:1000; anti-mouse IgG, #7076S, Cell Signaling Technology, 1:1000) were used in the experiments. Immunoblot analysis was performed as described previously (Ageta et al., 2018). Chemiluminescence was detected using ECL Plus (32132, Thermo Fisher Scientific) and signal was captured on an image analyzer LAS-4000 with Image Reader LAS-4000 (Ver2.0) and MultiGauge software (FUJIFILM).

### Statistical analysis

Statistical analyses were performed using Prism 9.4.1 (GraphPad Software). One-way ANOVA with Tukey's multiple comparison test (Figs 2 and 4) or Kruskal–Wallis with Dunn's test (Fig. 5) to compare three or more groups. Statistical methods, *P*-values, error bars, sample size, and the number of replicates are shown in the figure legends.
